# Increased production of inosine and guanosine by means of metabolic engineering of the purine pathway in *Ashbya gossypii*

**DOI:** 10.1186/s12934-015-0234-4

**Published:** 2015-04-17

**Authors:** Rodrigo Ledesma-Amaro, Ruben M Buey, Jose Luis Revuelta

**Affiliations:** Departamento de Microbiología y Genética, Metabolic Engineering Group, Universidad de Salamanca, Laboratory 323, Edificio Departamental, Campus Miguel de Unamuno, 37007 Salamanca, Spain

**Keywords:** Nucleoside fermentation, Metabolic engineering, Purine biosynthesis, *Ashbya gossypii*

## Abstract

**Background:**

Inosine and guanosine monophosphate nucleotides are convenient sources of the umami flavor, with attributed beneficial health effects that have renewed commercial interest in nucleotide fermentations. Accordingly, several bacterial strains that excrete high levels of inosine and guanosine nucleosides are currently used in the food industry for this purpose.

**Results:**

In the present study, we show that the filamentous fungus *Ashbya gossypii*, a natural riboflavin overproducer, excretes high amounts of inosine and guanosine nucleosides to the culture medium. Following a rational metabolic engineering approach of the *de novo* purine nucleotide biosynthetic pathway, we increased the excreted levels of inosine up to 27-fold.

**Conclusions:**

We generated *Ashbya gossypii* strains with improved production titers of inosine and guanosine. Our results point to *Ashbya gossypii* as the first eukaryotic microorganism representing a promising candidate, susceptible to further manipulation, for industrial nucleoside fermentation.

**Electronic supplementary material:**

The online version of this article (doi:10.1186/s12934-015-0234-4) contains supplementary material, which is available to authorized users.

## Background

Purine nucleotides are of significant economic interest for the applied biotechnology industry because they are used as foodstuff additives with flavouring, nutritional and pharmaceutical properties [1]. Inosine monophosphate (IMP) and guanosine monophosphate (GMP) have flavour enhancer capabilities that, in combination with monosodium glutamate, increase the umami flavour synergistically [2]. Moreover, both inosine and guanosine have beneficial health effects, related to their antioxidant, neuroprotective, cardiotonic and immunomodulatory properties [3-5]. It was estimated that approximately 22,000 tons of GMP and IMP were produced in 2010 [1].

Currently, purine nucleotides are obtained at industrial level mainly by microbial fermentation, either by RNA extraction and further breakdown into free nucleotides or by improved metabolic biosynthesis, subsequent excretion of the nucleosides to the culture medium, and further chemical or enzymatic phosphorylation [1]. In recent years, the significant enhancement of nucleoside production through metabolic engineering approaches in different microorganisms proved that this production process is the most efficient [6,7].

Most microorganisms synthesize purine nucleotides in two distinct pathways. First, purines are synthesized *de novo*, beginning with simple starting materials such as amino acids and bicarbonate. Alternatively, purine bases, either released by the hydrolytic degradation of nucleic acids and nucleotides or taken from the culture medium, can be recycled following the *salvage* pathway [8].

**Figure 1:**
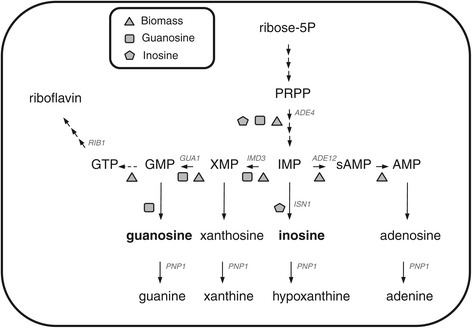
Simplified schematic representation of the *de novo* purine pathway in *A. gossypii*. Abbreviations used: *ADE4* phosphoribosylpyrophosphate (PRPP) amidotransferase, *ADE12* adenylosuccinate synthase, *IMD3* IMP dehydrogenase, *GUA1* GMP synthase and *PNP*1 purine nucleoside phosphorylase. The geometric shapes indicate the reactions for which the simulations using the genome-scale metabolic model iRL766 predicted an increase in the metabolic flux upon optimization of the production of biomass, guanosine and inosine (triangles, squares and pentagons, respectively).

In the de novo purine pathway, a molecule of ribose-5-phosphate is first converted through ten sequential catalytic reactions into IMP, the first compound in the pathway to have a completely formed purine ring system. IMP can then be converted into either AMP or GMP through the action of specific enzymes (Figure 1). Additionally, through the action of nucleotidases, nucleotides can be degraded into nucleosides that are either secreted to the culture medium or further degraded to nucleobases that can enter the salvage pathway (Figure 1; for a review, see [8,9]).

Several bacterial strains that overproduce nucleotides at industrial level have been developed by random mutagenesis and/or rational genetic modifications in *Bacillus subtilis* [10], *Bacillus amyloliquefaciens* [11], *Corynebacterium glutamicum* [12], *Corynebacterium ammoniagenes* [13] and *Escherichia coli* [7]. To date, metabolic engineering approaches to these bacteria have targeted: i) central metabolism ii) the *de novo* purine biosynthetic pathway 3) the *salvage* purine pathway 4) regulator genes and 5) nucleotide and nucleoside transporters [1].

*Ashbya gossypii* is a filamentous fungus considered to be a paradigm of the environmentally friendly “White Biotechnology” and is probably the most representative example illustrating the importance of microbial metabolic engineering to substitute chemical synthesis by a much more convenient microbial production. Contrary to what used to be the case several decades ago, at present most of the world’s riboflavin production relies on *A. gossypii* fermentation [14,15]. Additionally, it has one of the smallest genomes of free-living eukaryotes and a high level of similarity and gene order conservation (synteny) to the genome of the widely studied yeast *Saccharomyces cerevisiae* [16]. The existence of efficient gene targeting methods that allows the generation of stable engineered strains and a broad variety of molecular tools mean that *A. gossypii* is an ideal unicellular –eukaryotic- organism that is well suited for genetic manipulation and metabolic engineering [17-19]. In terms of industrial suitability, it has been found that *A. gossypii* can be readily scaled up for large-scale metabolite production [15]. Downstream processes are cheaper than in most yeast and bacteria because *A. gossypii* undergoes autolysis at low temperature and the mycelia can be removed by simple filtration [20]. Moreover, *A. gossypii* is able to use non-expensive waste products from other industrial processes as the sole carbon source [21]. Interestingly, the fact that *A. gossypii* is a natural riboflavin overproducer denotes a strong metabolic flux through the purine pathway (given that GTP is the riboflavin-limiting precursor) that could be redirected to accumulate nucleotides and nucleosides.

With all these advantages in mind, here we used a metabolic engineering approach to the purine pathway of *A. gossypii* aiming at generating strains with increased production levels of nucleosides of potential interest for the food biotechnological industry. In the present work, we demonstrate that the wild-type *A. gossypii* excretes high levels of the nucleosides guanosine and inosine to the culture medium, while their respective intracellular concentrations remain much lower. Furthermore, after manipulation of the genes that code for the enzymes in the *de novo* purine pathway, we generated strains with a 27-fold higher titer of excreted inosine than the wild-type, showing that *A. gossypii* is a promising eukaryotic candidate for industrial nucleoside production.

## Results and discussion

**Figure 2:**
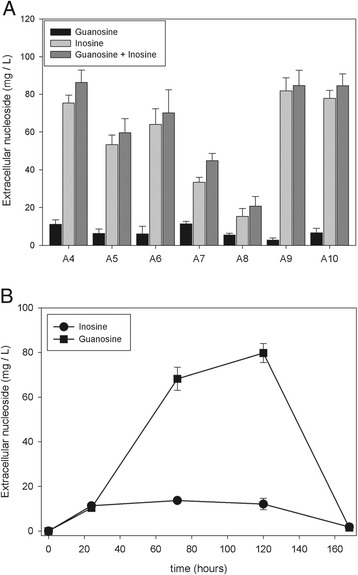
Quantification of inosine and guanosine in wild-type strains. **A**. Analysis of extracellular inosine and guanosine in different wild-type strains of *A. gossypii*. grown for five days in flasks containing MA2 rich medium. **B**. Time-course quantification of the excretion of inosine and guanosine to the culture media of strain **A4** grown in MA2. Error bars represent the standard deviations of three independent experiments.

*Inosine and guanosine nucleosides are excreted to the growth medium of A. gossypii.* We have recently generated a genome-scale metabolic model of *A. gossypii* [22] and have used it to investigate the transition from the initial growth phase to the late riboflavin-productive phase. Interestingly, in that work we observed nucleoside permeases to be significantly up-regulated during the latter stage [22]. Here, we aimed at further corroborating this prediction by analyzing the nucleosides excreted to the extracellular medium during flask fermentations of *A. gossypii*. As shown in Figure 2A, all the wild-type strains tested excreted mg/L amounts of nucleosides to the culture medium and these were almost exclusively guanosine and inosine. Based, on these results, we decided to use A4 (ATCC10895) as the parental strain for further modifications because: i) it is the strain that excretes the highest levels of nucleosides to the medium and ii) it is the best characterized wild type strain of *A. gossypii.*

A time-course analysis of A4 revealed a maximum in nucleoside excretion on the 5th day, rapidly falling off on around the 7th day of culture (Figure 2B); a similar type of behavior was also observed for the other strains tested (data not shown). These data suggest that the nucleosides excreted during the initial growth stages might be reincorporated to the cell, and then recycled to nucleotides through the salvage pathway, to support the strongly active riboflavin synthesis that occurs during the late stages of growth [22]. Alternatively, nucleoside degradation could occur in the culture medium, where cell lysis starts to take place at that stage [23]. However, these hypotheses should be corroborated in further experiments.

*Metabolic engineering of the purine pathway improves nucleoside excretion to the growth media of A. gossypii.* In order to determine the enzymes involved in nucleoside production, we used our computational genome-scale metabolic model [22] to simulate situations with three different objective functions; that is, to optimize the production of biomass synthesis, inosine or guanosine. A total of 303 reactions are involved in biomass production while only 76 and 78 respectively are predicted to maximize the production of inosine and guanosine (Additional file 1: Table S1). As expected, comparison of the three simulations revealed significant differences in the metabolic fluxes along the different reactions of the purine biosynthetic pathway, shown schematically in Figure 1. The flux through the genes upstream of IMP in the *de novo* pathway (*ADE4*, *ADE16*, etc.) was maximal in the three simulations, in contrast to *PNP1,* which was minimum. Moreover, the metabolic flux through *ADE12* decreased when the production of both inosine and guanosine was maximized. Additionally, the flux through ISN1 was maximized in simulations of inosine and guanosine production. On the other hand, our simulations predicted that the flux through *IMD3* should be maximized to optimize guanosine and minimized for inosine production (Figure 1).

Based on these computer simulations, and considering a predicted maximum flux as a target reaction/gene for overexpression and a minimum flux as a target reaction/gene for deletion, we next employed a rational metabolic engineering approach to generate *A. gossypii* strains with improved production titers. The genotypes of all the strains generated in this work and their respective nucleoside excretion levels are listed in Table 1.

**Table 1:** **Excreted nucleoside concentrations of the strains used in this work**

**Strain**	**Genotype**	**Parental strain**	**Inosine (mg/L)**	**Guanosine (mg/L)**
**A4**	Wild type	-	11.1 ± 2.4	75.3 ± 4.2
**A5**	Wild type	-	6.3 ± 2.3	53.3 ± 5.1
**A6**	Wild type	-	6.0 ± 4.2	64.1 ± 8.2
**A7**	Wild type	-	11.4 ± 1.3	33.4 ± 2.7
**A8**	Wild type	-	5.4 ± 1.0	15.3 ± 4.2
**A9**	Wild type	-	2.8 ± 1.1	81.7 ± 7.1
**A10**	Wild type	-	6.6 ± 2.3	77.8 ± 4.1
**I0**	^*^ *GPD:ADE4* ^*des*^	**A4**	12.5 ± 2.0	77.2 ± 6.1
**I1**	*GPD:ADE4* ^*des*^ *, ΔADE12*	**I0**	144.1 ± 7.3	92.8 ± 8.5
**I2**	*GPD:ADE4* ^*des*^ *, ΔADE12, ΔIMD3*	**I1**	227.9 ± 9.1	70.9 ± 6.2
**I3**	*GPD:ADE4* ^*des*^ *, ΔADE12, ΔIMD3, GPD:IMD3*	**I2**	44.51 ± 6.3	137.1 ± 3.4
**I4**	*GPD:ADE4* ^*des*^ *, ΔADE12, ΔPNP1*	**I1**	268.8 ± 8.1	138.0 ± 6.9
**I5**	*GPD:ADE4* ^*des*^ *, ΔADE12, GPD:ISN1*	**I1**	145.2 ± 5.1	92.2 ± 7.1
**I6**	*GPD:ADE4* ^*des*^ *, ΔADE12, ΔIMD3, ΔPNP*	**I4**	76.3 ± 4.8	27.1 ± 4.3
**I7**	*GPD:ADE4* ^*des*^ *, ΔADE12, GPD:GUA1*	**I1**	143.7 ± 6.2	91.3 ± 4.5
**I8**	*GPD:ADE4* ^*des*^ *, ΔADE12, RIB7p:RIB1*	**I1**	136.4 ± 8.2	100.0 ± 4.1

^*^This strain has been described previously in [17].

**Figure 3:**
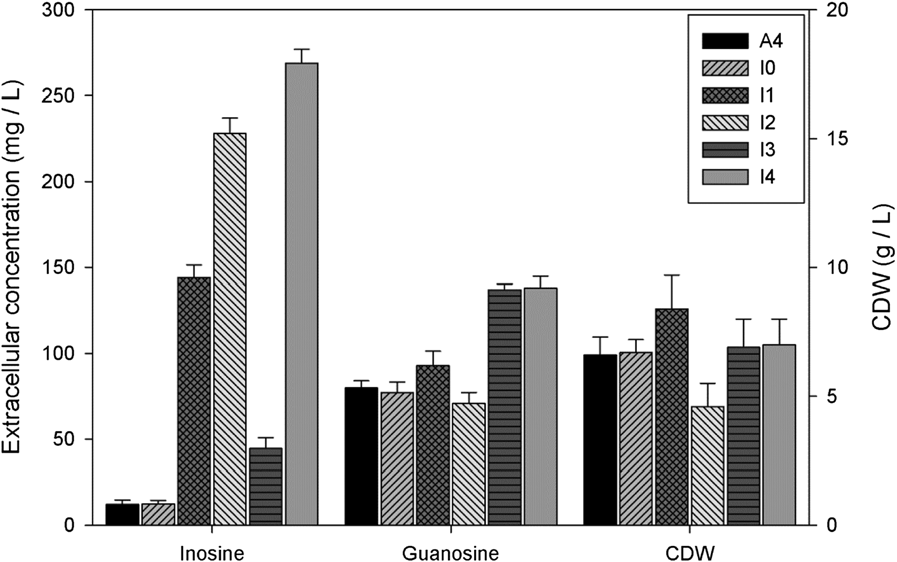
Extracellular concentrations of inosine and guanosine. Cultures of *A. gossypii* were grown for five days in flasks containing MA2. Error bars represent the standard deviations of three independent experiments.

As a first approach, we tested the nucleoside excretion levels of an engineered strain (I0) in which the metabolic flux through the purine pathway had been increased by overpassing the adenine-mediated transcriptional repression of the *ADE4* gene by eliminating the ATP/GTP feedback inhibition of the enzyme that it encodes, PRPP amidotransferase. We have previously demonstrated that increased metabolic flux through the purine pathway in this mutant raises riboflavin production significantly [17]. In fact, increased nucleoside production levels brought about by the deregulation of this enzyme in *E. coli*, *C. glutamicum* and *B. subtilis* have also been reported [6,7,12]. We therefore used this strain (I0) as the starting point for further genetic modifications. As shown in Figure 3 and Table 1, I0 excreted inosine and guanosine at similar levels to A4, indicating that most of the excess metabolic flux through the purine pathway is directed to riboflavin [17].

With the purine pathway enhanced in the I0 strain, we next attempted to further raise the flux through the guanine pathway to the detriment of the adenine pathway. To accomplish this, we depleted the *ADE12* gene that codes for adenylosuccinate synthase, the first enzyme in the adenine nucleotide pathway after the branch point between the GMP and AMP pathways (Figure 1). The resulting strain (I1) was auxotrophic for adenine but was able to grow at normal rates in rich medium and excreted about 13 times more inosine and 1.2 times more guanosine than A4 (Figure 3 and Table 1). These data suggest that the excess of IMP is, to a greater extent, converted to inosine, which is subsequently excreted to the medium and, to a lesser extent, directed to the guanine pathway and finally converted to riboflavin. Accordingly, I1 produces 2.5-fold higher amounts of riboflavin than A4 (data not shown).

We next disrupted the gene *IMD3* in the I1 strain to generate I2. *IMD3* encodes IMP dehydrogenase, the first enzyme in the guanine pathway after the branch point between the GMP and AMP pathways. I2 is auxotrophic for adenine and guanine and its growth rate is reduced even in rich medium. By blocking both the adenine (Δ*ADE12*) and the guanine (Δ*IMD3*) pathways, excess IMP is converted into inosine, as demonstrated by an almost 20-fold increase in the levels of excreted inosine of I2 with respect to A4 (Figure 3 and Table 1), even though the dry weight of I2 was lower than that of A4, due to its reduced growth rate. As expected, the excreted guanosine levels were lower in I2 than in A4 (Table 1).

We next attempted to direct the excess IMP into the guanine nucleotide pathway by overexpressing the *IMD3* gene (encoding IMP dehydrogenase), aiming to increase guanosine excretion levels. To accomplish this, we introduced the *IMD3* gene into I1, under the control of the strong constitutive glyceraldehyde 3-phosphate dehydrogenase (GPD) promoter, generating strain I3. As expected, I3 recovered prototroph for guanine and the excreted guanosine levels increased by about 2-fold, as compared to A4, to the detriment of inosine (Figure 3 and Table 1).

We also constructed a different strain, I4, in which the gene *PNP1* -which encodes the enzyme purine-nucleoside phophorylase- was disrupted to prevent the transformation of nucleosides into their respective nucleobases. I4 respectively excreted approximately 24- and 2-fold more inosine and guanosine than A4 (Figure 3 and Table 1).

Finally, we generated a strain, I5, in which the *ISN1* gene -encoding the enzyme IMP-specific 5′-nucleotidase- was overexpressed in order to enhance nucleoside production from the respective nucleotide. However, I5 did not show any significant increase in nucleoside excretion, indicating that the ISN1 gene does not limit nucleoside production in *A. gossypii*.

We next introduced the three modifications that most increased nucleoside levels in the culture medium into I0, i.e. *ΔADE12*, *ΔIMD3* and Δ*PNP1*, to generate strain I6. This strain exhibited a rather limited growth pattern even in rich medium (data not shown). Nonetheless, even though the total levels of excreted nucleosides per liter of culture were lower than for the other strains (Figure 3 and Table 1), I6 showed the best production titers per cell dry weight (85- and 3-fold more inosine and guanosine than A4 respectively). In fact, when hypoxanthine, guanine and adenine were added to the culture medium, I6 almost recovered wild-type growth rates and excreted high amounts of nucleosides; despite this, these amounts were still below those ones observed for I4 (Additional file 2: Figure S1). Accordingly, we did not further consider this strain as a potential biotechnological tool.

In sum, by using a classical metabolic engineering approach that targeted purine nucleotide biosynthesis we obtained *A. gossypii* strains in which inosine excretion to the culture medium was significantly improved, and increased 25-fold with respect to the wild-type strain A4. The most favourable modifications included the overexpression of a deregulated *ADE4* mutant gene and the deletion of *ADE12* plus either *IMD3* (I2) or *PNP1* (I4). The combinations of all four mutations simultaneously afforded strains with compromised cell viability, and hence were not useful for industrial use.

*The increased metabolic flux through the de novo purine nucleotide pathway is mostly directed to inosine excretion in the mutant strains.* We further determined the intracellular levels of the nucleotides, nucleosides and nucleobases that participate in the purine biosynthetic pathway, as shown in Figure 1 of A4, I1, I2 and I4, since these are the wild-type and the strains with the highest biotechnological potential. This would not only allow us to further explore the effects of the mutations on the purine pathway but also to identify potential bottlenecks susceptible to manipulation in order to increase nucleoside production.

**Figure 4:**
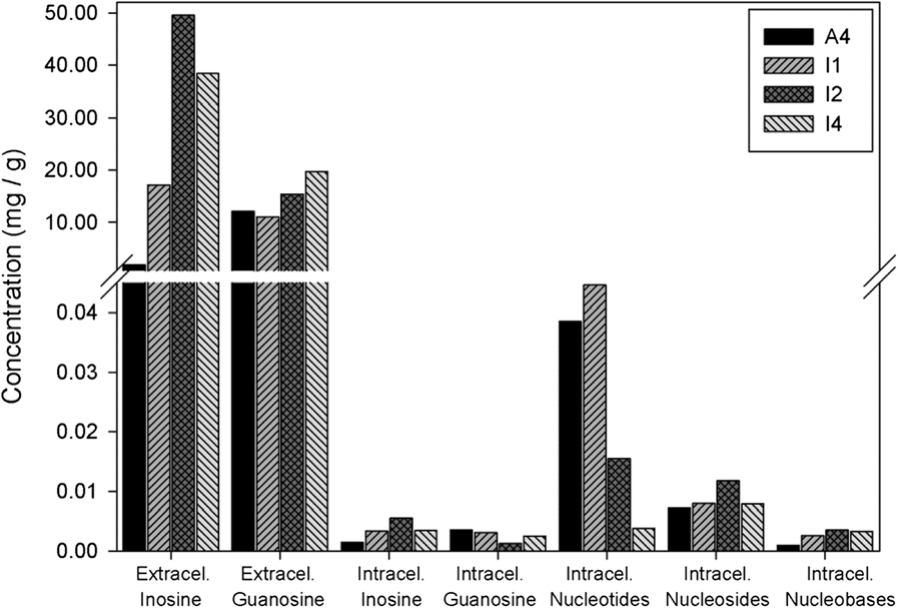
Extracellular and intracellular purines in A. gossypii engineered strains. Intracellular concentration of nucleotides (IMP, XMP, GMP and AMP), and extracellular concentration of nucleosides (inosine, xanthosine and guanosine) and nucleobases (hypoxanthine, xanthine, guanine and adenine) in *A. gossypii* engineered strains.

**Figure 5:**
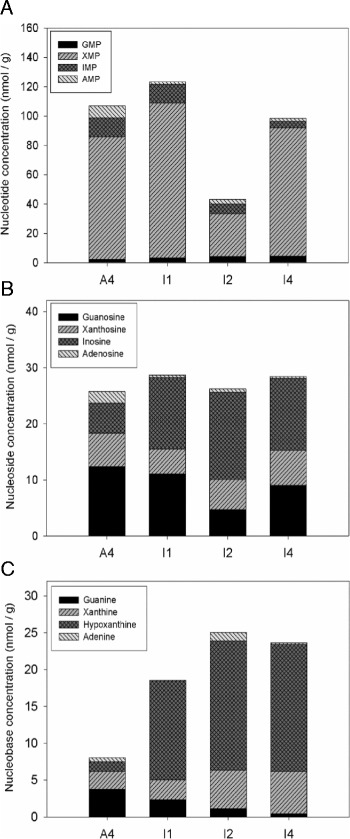
Intracellular metabolites of the purine pathway in strains A4, I1, I2 and I4. **A**. GMP, XMP, AMP and IMP nucleotide concentrations. **B**. Guanosine, xanthosine, adenosine and inosine nucleoside concentrations. **C**. Guanine, xanthine, adenine and hypoxanthine nucleobase concentrations.

Intriguingly, the total nucleoside (guanosine, xanthosine, inosine and adenosine) intracellular levels, in the nmol/g range, were about three orders of magnitude lower than the extracellular ones (Figure 4). Moreover, the variations in extracellular nucleoside levels between the wild-type and engineered strains (Figure 3) were significantly more marked than the corresponding variations in the intracellular metabolites (Figure 5).

In the wild-type strain, A4, guanosine levels were higher than those of inosine, in accordance with the excreted nucleosides in A4 (Figure 2A). As expected, *ade12* depletion (strains I1, I2 and I4) redirected the purine metabolic flux towards inosine/GMP at the expense of AMP production, as reflected in a marked decrease in AMP and adenosine intracellular levels in these strains (Figure 5). Remarkably, the excess IMP generated in these strains was preferentially directed to inosine and hypoxanthine rather than to GMP/guanosine production (Figure 5). This was subsequently reflected in the respective nucleoside extracellular levels: while a marked increase in excreted inosine was observed in the mutant strains studied, only subtle changes in excreted guanosine were observed (Figure 3 and Table 1).

Thus, the concentration of inosine in the culture medium of *A. gossypii* flask fermentations is proportional to the metabolic flux through the purine pathway, representing a very convenient methodological approach for determining relative differences in metabolic engineering approaches.

Interestingly, all strains, except I2 (where *IMD3* was disrupted), accumulated intracellular XMP, suggesting either a tight regulation at this level or a low efficiency of the enzyme GMP synthase. To overcome this, we used I1 as the parental strain to construct I7, in which the *GUA1* gene -encoding the enzyme GMP synthase- was overexpressed by placing it under the control of the strong constitutive promoter of GPD. However, I7 did not show higher levels of excreted guanosine (Table 1) but instead increased the production of riboflavin (data not shown), indicating that most of the excess metabolic flux through the guanine nucleotide pathway was directed beyond GMP/GTP to riboflavin synthesis, as has been previously reported by our lab [17,18]. We thus decided to down-regulate riboflavin synthesis by placing the *RIB1* gene (which catalyzes the first and one of the limiting steps of riboflavin biosynthesis) under the control of the *RIB7* promoter, generating strain I8. The *RIB7* promoter has been shown to produce low transcription levels and can therefore be used to down-regulate gene expression in *A. gossypii* (Ledesma-Amaro et al., submitted manuscript). Indeed, this strain showed decreased riboflavin production (not shown), as expected, but guanosine excretion did not increase significantly (Table 1).

Taken together, our data show that the excess metabolic flux through the purine pathway in *A. gossypii* is mostly derived to produce either riboflavin (in the late growth phase) or inosine (in the mid-early growth phase), which are subsequently excreted to the culture medium. Accordingly, inosine excretion might act as a mechanism to prevent an overflow through the guanine nucleotide pathway that would alter the guanine nucleotide pool and compromise cell viability.

To conclude, we have constructed *A. gossypii* strains with improved inosine excretion levels up to 0.27 g/L in culture media containing 20 g/L of glucose. Moreover, as an initial approach we have only focused on the systematic manipulation of the *de novo* purine biosynthetic pathway, but it is envisaged that additional manipulations of the central metabolism, global metabolic regulators, the *salvage* pathway, as well as transporters, will improve nucleoside excretion, as described for other organisms [10]. In any case, although further strain manipulation and optimization of the culture medium composition and fermentation conditions are required, our results demonstrate that *A. gossypii* is a promising candidate for use in the production of nucleosides at industrial scale. In fact, the nucleoside production titers described in this work are comparable to those of riboflavin (10–100 mg/L) when grown in flasks containing MA2 rich medium but *A. gossypii* wild-type strains are able of produce up to 5.5 g/L of riboflavin after partial optimization of the medium composition [24]. Likewise, higher nucleoside production titers are envisaged after systematic optimization of large-scale fermentation conditions in *A. gossypii*.

To the best of our knowledge, this work represents the first description of an eukaryotic organism for potential use in industrial nucleoside production. It should be remarked here that *A. gossypii* presents several advantages with respect to the bacterial systems currently used in industry: i) the convenience of the filtration/centrifugation processes to separate the biomass from the culture medium owing to the much larger size of the mycelia with respect to bacterial cells, ii) *A. gossypii* barely excretes any extracellular fermentative by-products; accordingly, nucleoside-enriched medium could be readily separated from the mycelium by simple and non-expensive filtration and/or centrifugation procedures, facilitating downstream processing to a considerable extent and iii) the availability of protocols for large-scale fermentation already implemented for riboflavin production.

## Conclusions

Using a rational metabolic engineering approach, aided by computational predictions, we have obtained *Ashbya gossypii* strains with improved nucleoside production titers that are very promising candidates for further optimization and use in nucleoside production at industrial level.

## Methods

### *A. gossypii* strains, media, and growth conditions

All the *A. gossypii* strains described in this manuscript are listed in Table 1. They were cultured at 28°C using MA2 rich medium [25]. Transformation, sporulation conditions and spore isolation were as described in detail elsewhere [26]. Gene disruption and overexpression were performed as previously published [17,20,27]. Gene down-regulation was achieved by placing the desired gene under the control of the *RIB7* promoter, which ensures low transcription levels (Ledesma-Amaro et al., submitted manuscript). Correct DNA integration was corroborated by sequencing for all the strains used in this study.

#### Metabolite determination

Mycelia from 5 mL culture broth were harvested by filtration on filter paper, dried overnight at 100°C and weighed. Filtered medium was passed through a 0.2 μm PVDF membrane (Acrodisc® LC; Pall, Life Sciences) and injected into an AQUASIL C18 140×4.6 mm column (Thermo Scientific) connected to an HPLC device (Agilent 1120 Compact LC) to determine extracellular nucleoside concentrations by monitoring absorbance at 260 nm. The separation of nucleosides was achieved by using an isocratic flow of phosphate buffer, pH 5.5, plus 0.5% of acetonitrile. Quantification was carried out using a calibration curve prepared with pure standards of inosine and guanosine (Sigma-Aldrich). All analyses were performed using three biological replicates.

Metabolism quenching and metabolite extraction were carried out as described [28,29], using the mycelia obtained from 7 mL of culture broth. Briefly, samples were quenched by the addition of methanol at −40°C, followed by boiling ethanol, and extracted upon the addition of acetonitrile by mechanical homogenization and subsequent chloroform/chloromethane organic extraction steps. The intracellular concentrations of the metabolites in the purine pathway were determined following previously published methodologies [30].

## Additional files

Additional file 1: Table S1.
**Reaction carrying flux according to the genome scale metabolic model iRL766.** Column A represents the reaction carrying flux when the production of biomass is maximized. Column E and I represents the reaction carrying flux when the production of inosine and guanosine are respectively maximized. Additional file 2: Figure S1.
**Extracellular nucleosides in the strain I6.** A. Extracellular concentrations of inosine and guanosine in mg per liter of culture. B. Extracellular concentrations in mg of inosine and guanosine per gram of CDW. Cultures were grown for five days in flasks containing MA2. Where indicated, hypoxanthine, guanine and adenine nucleobases at a final concentration of 150 μM were added to the media. Error bars represent the standard deviations of three independent experiments.
