# Supplementary figures and images for: Increased production of inosine and guanosine by means of metabolic engineering of the purine pathway in Ashbya gossypii
Source: Microb Cell Fact. 2015 Apr 17;14:58. doi: 10.1186/s12934-015-0234-4 (PMC4407346; doi:10.1186/s12934-015-0234-4)

## Slide 1
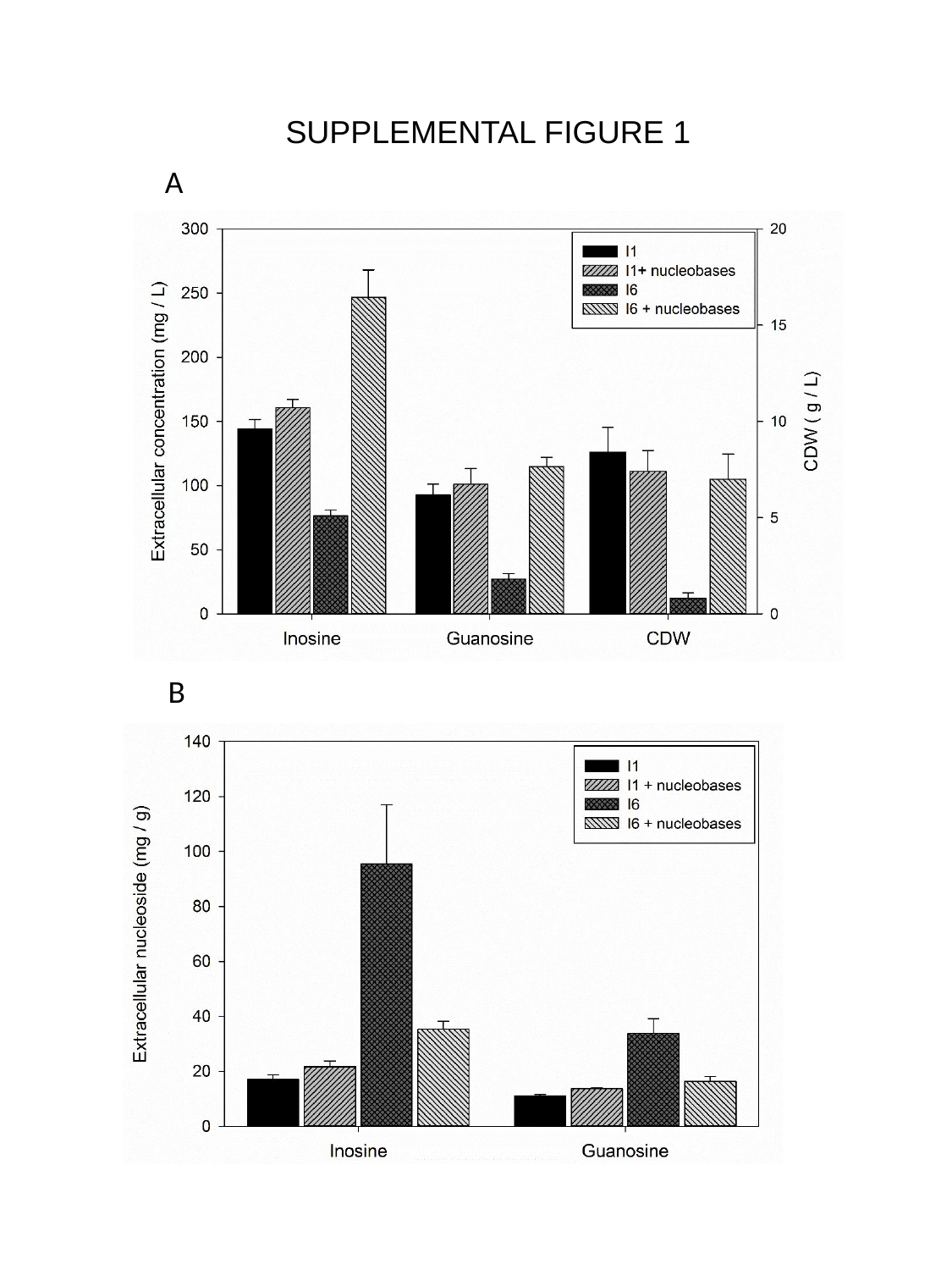

SUPPLEMENTAL FIGURE 1
A
B

Supplement: Additional file 2: Figure S1. — Extracellular nucleosides in the strain I6. A. Extracellular concentrations of inosine and guanosine in mg per liter of culture. B. Extracellular concentrations in mg of inosine and guanosine per gram of CDW. Cultures were grown for five days in flasks containing MA2. Where indicated, hypoxanthine, guanine and adenine nucleobases at a final concentration of 150 μM were added to the media. Error bars represent the standard deviations of three independent experiments. [file 12934_2015_234_MOESM2_ESM.ppt]
